# Overweight and Obesity among Recipients of Antiretroviral Therapy at HIV Clinics in Gaborone, Botswana: Factors Associated with Change in Body Mass Index

**DOI:** 10.1155/2020/8016791

**Published:** 2020-01-04

**Authors:** Jose Gaby Tshikuka, Mgaywa Gilbert Mjungu Damas Magafu, Goabaone Rankgoane-Pono, Julius Chacha Mwita, Tiny Masupe, Shimeles Genna Hamda, Roy Tapera, Mooketsi Molefi, Joseph Tshibangu, John Thato Tlhakanelo

**Affiliations:** ^1^Department of Family Medicine and Public Health, Faculty of Medicine, University of Botswana, Gaborone, Botswana; ^2^Department of Health Sciences, National Pedagogic University, Kinshasa I, Democratic Republic of the Congo; ^3^Disease Intelligence and Surveillance Division, Africa Centres for Disease Control and Prevention (Africa CDC), African Union Commission, Addis Ababa, Ethiopia; ^4^Department of Internal Medicine, Faculty of Medicine, University of Botswana, Gaborone, Botswana; ^5^School of Public Health, Faculty of Health Sciences, University of Botswana, Private Bag 0022, Gaborone, Botswana

## Abstract

**Background:**

Factors associated with overweight/obesity among antiretroviral therapy (ART) recipients have not been sufficiently studied in Botswana.

**Objectives:**

To: (i) estimate the prevalence and trends in overweight/obesity by duration of exposure to ART among recipients, (ii) assess changes in BMI categories among ART recipients between their first clinic visit (BMI-1) and their last clinic visit (BMI-2), (iii) identify ART regimen that predicts overweight/obesity better than the others and factors associated with BMI changes among ART recipients.

**Methods:**

A 12-year retrospective record-based review was conducted. Potential predictors of BMI change among patients after at least three years of ART exposure were examined using a multiple logistic regression model. Adjusted odds ratios (AOR) and their 95% confidence intervals (CIs) were computed. ART regimens, duration of exposure to ART, and recipients' demographic and biomedical characteristics including the presence or absence of diabetes mellitus-related comorbidities (DRC), defined as any morbidity associated with type 2 diabetes as described in the international statistical classification of diseases and related health problems (ICD-10-CM) codebook index, were investigated as potential predictors of overweight/obesity.

**Results:**

Twenty-nine percent of recipients were overweight, 16.6% had obesity of whom 2.4% were morbidly-obese at the last clinic visit. Overweight/obese recipients were more likely to be female, to have DRC and less likely to have CD4 count between 201 and 249 cells/mm^3^. Neither the first-line nor the second-, third-line ART regimens predicted overweight/obesity better than the other and neither did the duration of exposure to ART. No significant linear trends were observed in the prevalence of overweight/obesity by the duration of exposure to ART.

**Conclusion:**

These results suggest that the ART regimens studied have a comparable effect on overweight/obesity and that the duration of exposure does not affect the outcome. This study calls for further research to elucidate the relative contribution of various factors to BMI change among recipients, including ART regimens.

## 1. Introduction

The advent of ART has changed the clinical picture of HIV/AIDS. Wasting syndrome, one of the WHO HIV/AIDS severity classification criteria, is now less common among PLWH [1–3]. Overweight/obesity has become more common [4, 5]. Some experts considered this shift of HIV/AIDS clinical picture or the weight gain among PLWH as a side effect of all ART regimens [6], while for others, it was considered to be an immunological response or a reflection of an increased CD4 cell count [7]. This is because of the substantial number of reports of associations between CD4 cell increase and weight gain [7, 8]. Protease inhibitors (PI), for instance, have been associated with weight gain, mainly with fat mass, condition like buffalo syndrome and increased central body fat distribution similar to metabolic syndrome, with no change in lean body mass [8, 9]. However, a study by Todd and colleagues [10] unearthed associations between Nucleoside Reverse Transcriptase Inhibitors (NRTI) and increased odds of hyperinsulinemia, whereas cumulative exposure to Non-Nucleoside Reverse Transcriptase Inhibitors (NNRTI) or PI drugs lacked association with insulin resistance markers or correlates of DRC and overweight/obesity. Recent work by Obry-Roguet and co-workers [11] failed to identify any association between all ART combinations and overweight/obesity. Their results partly support previous reports by Hasse and colleagues [12] that only limited associations existed between ART and overweight/obesity. These conflicting reports are confusing and make it difficult to consider all cases of overweight/obesity as side effects of ART. The real contribution of ART on BMI change is therefore challenged and needs to be well defined. So far, only the restoration of recipients' health status through viral load suppression and CD4 cell increase attributed to ART is an indisputable fact [13–15]. The weight gain following sustained viral load suppression may be explained by multiple factors of which ART might not be a significant direct contributor. This is because at this particular stage PLWH can gain weight just as the general population. Nutritional, sociodemographic, economic, biomedical, genetic, psychological, emotional, and behavioural factors like alcohol, tobacco, and substance use have all been associated with BMI change [11, 16]. These factors cannot be ignored when investigating the effects of ART on BMI change. The identification of such factors is important for effective interventions to improve the quality of life of PLWH.

Sub-Saharan Africa is currently experiencing an epidemiological transition with a growing number of non-communicable diseases (NCDs), particularly overweight/obesity, due to the decline in HIV/AIDS morbidity and mortality owing to easy access to ART [17]. Botswana is one of the most HIV/AIDS-affected countries in the world. It is also one of the first countries to implement a free and comprehensive ART program. Thus, ART is widely used by PLWH in this country. The ART is administered in different regimens [18]. The question is, which of the regimens induce(s) overweight/obesity more than the others? Also, how long does it take for this undesirable outcome to occur? Answers to these questions do not exist, yet they are central to an effective clinical management program for PLWH. The objectives of this study were to: (i) estimate the prevalence and trends in overweight/obesity by duration of exposure to ART among recipients, (ii) assess changes in BMI categories among ART recipients between their first clinic visit (BMI-1) and their last clinic visit (BMI-2), and (iii) identify the ART regimen which predicts overweight/obesity better than the others and elucidate factors associated with BMI changes among ART recipients.

## 2. Methods

### 2.1. Operational Case Definitions

In this study, overweight/obesity was defined as the aggregation of overweight and all categories of obesity. Overweight, obesity and other BMI categories were each defined as by the US National Institutes of Health [19].

Diabetes-related comorbidity (DRC) was defined as any morbidity associated with type 2 diabetes as defined in the ICD-10-CM codebook index [20]. Thus, patients diagnosed by the attending physician with hypertension/high blood pressure, lipodystrophy/lipoatrophy, renal dysfunction, cardiovascular conditions, low-density lipoprotein cholesterol (LDL-C), or their combinations were considered as having DRC.

### 2.2. Study Area and Design

The study was a retrospective record-based review of HIV patients in Gaborone, Botswana. Data from 2002 to 2015 were collected at two HIV clinics, namely Princess Marina Hospital ART Clinic and Bontleng ART Clinic.

### 2.3. Sampling Strategy

Client record numbers from both clinics were used to form the sampling frame. A computer table of random numbers was used to select 540 patients. Patients were excluded or included in the study based on consistency of their data in clinic admission or follow up registers, files, discharge registers, and referral registers. Patients were excluded if they had been clinically diagnosed with DRC by the attending physician at the time of entry into the study or first clinic visit. Also excluded were pregnant women, patients who were initiated on ART regimen after the year 2012 (allowing for at least three years of exposure), patients aged less than 18 years and those with unmatched data from different records within the same facility.

### 2.4. Data Collection

Data were extracted from patient records and the following variables collected: age at the first clinic visit (age-1), gender, date of enrolment into the ART program, date of initiation on ART, weight (in kilograms) at the first clinic visit (weight-1) and height (in centimetres), weight (in kilograms) at the last clinic visit (after exposure to ART) (weight-2), CD4 cell count at the first clinic visit (CD4-1), CD4 cell count at patients last clinic visit (CD4-2), presence or absence of DRC, ART regimen (first-, second- or third-line) and name of clinic attended.

### 2.5. Data Analysis

Data were analysed using IBM SPSS version 25 (Chicago, IL). Recipients' age-1 was estimated in years. Age at the last clinic visit (Age-2) was computed by adding the number of months of exposure to ART to age-1. Patients diagnosed by the attending physicians as having “hypertension” and those diagnosed as having “high blood pressure” were combined in one group of “hypertension” during the analysis.

ART regimens were categorized as defined by the Botswana National HIV & AIDS Treatment Guidelines [21] and the Handbook of the Botswana Integrated HIV Clinical Care Guidelines [22] in use between 2002 and 2015. The first-, second- and third-line regimen details are as reported by Rankgoane-Pono et al. [18] and in Table 1.

ART recipients' BMIswere estimated by dividing the weight (in kilograms) by the square of the height (in meters) at the first clinic visit (BMI-1) and at the last clinic visit (BMI-2). Patients with BMI <18.5 kg per m^2^ were classified as underweight, those with BMI between 18.5 and 24.9 kg per m^2^ were classified as having normal BMI, those between 25.0 and 29.9 kg per m^2^ were considered as overweight, those between 30.0 and 39.9 kg per m^2^ had obesity and those with BMI ≥40.0 kg per m^2^ were considered as morbidly obese. For analysis purposes, overweight, obesity, and morbidly obese participants at the last clinic visit were aggregated as a single group “overweight/obesity”.

Descriptive analysis was performed to characterize patients according to their BMI. Patients with DRC were identified based on the attending physician's diagnosis and according to ICD-10 coding. The duration of exposure to ART was estimated by computing the time difference between the date of the last clinic visit and the date of initiation on ART, expressed in months. Prevalence of BMI categories among patients was estimated by dividing the number of cases in each group by their respective sample, then multiplied by 100. The prevalence of overweight/obesity was plotted against the duration (months) of exposure to ART. Chi-square for linear trends was estimated and a trends line was fitted. To assess changes in BMI among ART recipients, patients' BMI at the first clinic visit (BMI-1) was compared with their BMI at the last clinic visit (BMI-2) using the McNemar test.

Demographic and biomedical factors associated with overweight/obesity were investigated among the patients at the first and last clinic visits. Group/subgroup comparisons for continuous variables were performed using the Kruskal–Wallis test for quantitative variables or ANOVA. Unadjusted odds ratios (UORs) and their 95% CI were estimated for categorical variables.

To identify the ART regimen which predicts overweight/obesity better than the others and factors associated with BMI changes among ART recipients, a multivariate logistic regression model was run to compute adjusted odds ratios (AOR) and associated 95% CI. The following were investigated as potential exposure variables: ART first-line or second-, third-line regimens, duration of exposure to ART in months (as a continuous variable), age-2 (as a continuous variable), gender, CD4-2 cells/mm^3^ (as categorical variable: 0–200, 201–349, and ≥350 cells/mm^3^) and the presence or absence of DRC. Other variables such as HCV status, alcohol and substance abuse were missing from the hospital records and were not investigated. All statistically significant variables and some variables which were not statistically significant but of interest were kept in the model to measure the relative contribution of each of them to the outcome of interest.

Variables suspected to have different relationships with the outcome variable depending on the third factor were investigated in a series of interaction terms. The level of significance was set at *p* < 0.05. The Cox and Snell *R*^2^ was estimated and how well the data fitted the model was investigated by computing the Hosmer and Lemeshow *p*-value.

### 2.6. Ethical Considerations

Ethical approval to collect data was sought and obtained from the University of Botswana Review Board and the Ethics Committee of the Ministry of Health and Wellness in Botswana. Permission to collect the data from clinic record books and electronic systems was also sought and obtained from relevant clinic management.

## 3. Results

### 3.1. Patient Characteristics

Patient characteristics and outcomes of bivariate analysis by BMI-1 classified as underweight, normal, and overweight/obesity at first clinic visit or before ART initiation are presented in Table 2. No significant differences were observed between the mean ages of the three BMI subgroups (*p* = 0.520). Female patients were more likely to be overweight or to be obese compared to their male counterparts (UOR = 2.64, *p* = 0.001). Underweight patients had the lowest median CD4 cell count, followed by recipients with normal BMI, while patients with overweight/obesity had the highest median CD4 cell count (*p* < 0.030). The risk of being overweight or having obesity in patients with CD4 nadir of 0–200 cells/mm^3^ was 76% less than that of patients who had CD4 cell count ≥350 cell/mm^3^ (UOR = 0.24, *p* < 0.001), while in those with CD4 cell count of 201–349 cell/mm^3^it was 67% less than that of patients who had CD4 cell count ≥350 cell/mm^3^ (UOR = 0.33%, *p* < 0.001). Nine patients (1.7%) of the total number recruited had DRC at the commencement of the study and were excluded from it.

Of the 114 patients who were overweight/obese at their first clinic visit, 48 (42%) reverted to normal BMI at the last clinic visit, 2 (1.8%) became underweight. Data presented in Table 3 show participants' BMI status at the first clinic visit BMI-1 and the last clinic visit BMI-2. Significant differences were noticed between participants' BMI-1 and BMI-2 (*p* < 0.001).

Patient characteristics after initiation on ART or at their last clinic visit by BMI-2 category recorded as underweight, normal, and overweight/obesity are presented in Table 4. No significant differences were noticed between the mean age of the three BMI categories (*p* = 0.180). None of the ART regimens in use was associated with the recipients' overweight/obesity status more/less than the other (UOR = 1.2, *p* = 0.250). The duration of exposure to these ART drugs was not associated with overweight/obesity among recipients (*p* = 0.190). Female recipients were more likely to be overweight and to have obesity compared to their male counterparts (UOR = 3.0, *p* = 0.001). Underweight recipients had a median [(interquartile range (IQR)] CD4 cell count of 444 (270–597) cells/mm^3^, recipients with normal BMI had a median (IQR) CD4 cell count of 513 (375–686) cells/mm^3^ while overweight/obesity recipients had a median (IQR) CD4 cell count of 577 (416–732) (*p* = 0.001).

Recipients with CD4 cell count nadir of 0–200 cells/mm^3^and those with CD4 cell count of 201–349 cells/mm^3^were less likely to be overweight/obese compared to those with CD4 cell count of ≥350 cells/mm^3^ (UOR = 0.29, *p* = 0.003, and UOR = 0.27, *p* = 0.001, respectively). Recipients who had DRC had an 88% higher risk of being overweight/obese than those without DRC (UOR = 1.88, *p* = 0.007).

At the last clinic visit, 89 (16.8%) recipients were diagnosed with DRC. Of them, 24 (26.9%) were diagnosed as hypertensive, 20 (22.5%) had lipodystrophy/lipoatrophy, 6 (6.7%) had renal dysfunction, 7 (7.9%) had cardiovascular conditions, 11 (12.4%) had LDL-C and 21 (23.6%) had different combinations of these conditions.

**Figure 1:**
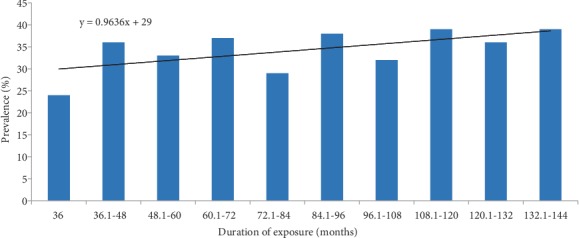
Prevalence (%) and trends in overweight/obesity among ART recipients from Princess Marina Hospital and Bontleng anti-retroviral therapy clinics in Botswana (*N* = 531).

The overall prevalence of overweight alone was 28.8%, while obesity was prevalent in 16.6% of the recipients. Overweight or obesity was prevalent in 45.4% of recipients. No significant trends were observed in the outcome by the duration of exposure. Data presented in Figure 1 show relatively similar prevalence rates of overweight/obesity by duration (months) of exposure to ART expressed by a lack of significant linear trend of the outcome (*p* = 0.2). The average duration of exposure to ART was 85 months (a minimum of 36 months and a maximum of 144 months).

### 3.2. Predictors of Overweight/Obesity and Factors Independently Associated with Overweight/Obesity among ART Recipients at Their Last Clinic Visit

Multivariate logistic analysis was used to identify predictors and factors independently associated with overweight/obesity among patients after at least three years of exposure to ART. Results presented in Table 5 show that recipients with overweight/obesity were more likely to be females (AOR = 2.84; 95% CI: 1.83–4.42). Recipients with nadir CD4 count of 0–200 cells/mm^3^ were 70% less likely to be overweight or obese compared with recipients who had a CD4 count ≥350 cells/mm^3^ (AOR = 0.30; 95% CI: 0.17–0.55). Those with CD4 count 201–249 cells/mm^3^ were 62% less likely to be overweight or obese compared to those with a CD4 count ≥350 cells/mm^3^ (AOR = 0.38; 95% CI: 0.16–0.89). Recipients with DRC had a 2.2 times higher risk of developing overweight or obesity compared to those who did not have DRC (AOR = 2.2; 95% CI: 1.18–3.39). Neither the first-line nor the second-, third-line ART regimens predicted overweight/obesity better than the other (AOR = 1.22; 95% CI: 0.82–1.79). The duration of exposure to ART was not associated with the development of overweight/obesity among the recipients.

## 4. Discussion

This study reviewed medical records of 531 PLWH who attended two main HIV clinics in Botswana between 2002 and 2015. The study investigated overweight/obesity among recipients of ART and compared the effect of different ART regimens on overweight/obesity in this middle-income country. The overall prevalence of overweight alone among this group of PLWH was 28.8%, obesity alone was 16.6%, and overweight/obesity was 45.4%. These results look similar to those reported by another researcher on the general population of Botswana [16] despite being from two different subpopulations. This may suggest that rates of overweight/obesity between the two subpopulations are comparable. This assumption is strongly supported by research conducted in the US by Crum-Cianflone and coworkers [4, 5]. These authors reviewed data from two US Navy clinics and found no difference in the prevalence of overweight/obesity between recipients of ART and HIV-negative patients in the US. They concluded that the finding was not unexpected because of easy access to ART, a treatment that makes PLWH live normal lives and longer [4] eventually encountering the same health problems as the general population [23].

Botswana is one of the few countries which have made significant progress toward meeting the Joint United Nations' Program on HIV/AIDS (UNAIDS') targets by 2020, whereby 90% of all PLWH are expected to know their HIV status, 90% of whom are expected to receive sustained ART, and 90% of those on ART are expected to have virological suppression [24]. Thus, results showing overweight and obesity prevalence comparable to those of the general population may not be a surprise [16]. More importantly, is the fact that recipients who were overweight/obese were more likely to be female and less likely to have the nadir CD4 count of 0–200 or CD4 count of 201–249 cells/mm^3^ which corroborates the literature [16, 25]. ART suppresses viral replication and increases CD4 count resulting in the restoration of recipients' health status [25]. Thus, the BMI change observed among the recipients herein might result from a natural process or interplay of different factors [11, 15, 26]. By preventing the advancement of HIV infection, ART allows other bodily processes to proceed normally and increase the recipients' weight. This is supported by the significant changes observed in BMI indicators in the last clinic visit after ART initiation. The observation confirms the effect of initiation to ART on recipients' BMI change through suppression of viral replication and CD4 cell increase [25]. However, at the last clinic visit, not all the recipients gained weight within their normal BMI. Some recipients became overweight/obese, while some of those who were overweight/obese at baseline reverted to normal BMI or unexpectedly became underweight. Neither the first-line nor the second-, third-line ART regimens predicted overweight/obesity better than the other; and neither did the duration of exposure to ART. These observations cast doubt as to whether the overweight/obesity seen among these recipients is merely a side effect of ART. The reversion from overweight/obesity to normal BMI, for instance, is easy to comprehend as this might be a simple effect of regular physical exercise [26, 27]. On the other hand, it is hard to comprehend how overweight/obese recipients became underweight after ART initiation. One would have expected the BMI to increase rather than decrease after ART initiation if every case of BMI increase was truly due to ART side effects. The absence of differences between ART regimens in predicting overweight/obesity in this population does not mean that there is no association between ART and the outcome. This is well illustrated in this study by the significance of the difference between BMI-1 and BMI-2. The unanswered question here is how much ART contributes to overweight/obesity among the recipients. Further studies are needed to address this question for effective and tailored interventions to improve the quality of life of PLWH.

The 12% proportion of the variability of the outcome explained by the multivariate model [Cox and Snell *R*^2^ statistics = 0.12] is of particular interest given the number and types of variables in the model and calls for further exploration of other potential risk factors. Focussing only on biomedical and demographic factors is one of the limitations of this study. Overweight and obesity are known as primarily nutritional and socioeconomic corollaries [28] even though other factors such as biomedical, demographic and genetic have also been implicated [16, 28]. Although a study which includes all these factors in a single model would have been the best approach to identify predictors of the outcome under investigation, such a study is hard to find. The retrospective nature of our study makes it even more difficult to have all the factors investigated in one model. Despite this limitation and the lack of information on pregnancy during the follow-up period, variables identified here as correlates of overweight/obesity among ART recipients, namely CD4 count, gender and DRC deserve attention so as to minimize morbidity among ART recipients.

## 5. Conclusion

The prevalence of overweight/obesity among ART recipients is high in Botswana. Overweight/obese recipients are more likely to be female and more likely to have a high CD4 cell count. ART recipients experienced significant changes in their BMI over time. However, overweight/obesity did not vary with the duration of exposure to the ART. In the studied population, no ART regimen was found to have more propensity to affect BMI than the other. Further research is needed to elucidate the relative contribution of various factors to BMI change among recipients, including ART regimens.

## Figures and Tables

**Table 1:** Standard first- and second-line ART regimens in Botswana at the time of the study.

First-line regimen	First-line modifications	Second-line regimen	Second-line modifications
AZT + 3TC (CBV) + EFV	TDF renal toxicity without CVD risk: ABC/3TC/DTG (If rash: consult HIV specialist)	TDF + FTC + ALU	AZT anemia and/or TDF renal toxicity: ABC/3TC/DTG
AZT + 3TC + NVP			
AZT + DDI + EFV			
AZT + DDI + NVP			
	CNS toxicity and/or Hepatic toxicity: TRU/DTG		
TDF + FTC (or 3TC) + EFV		CBV + ALU	If anemic ABC + 3TC + ALU
TDF + FTC (or 3TC)+NVP			
D4T + 3TC + EFV		TDF + FTC = ALU	If renal insufficiency but no anemia: CBV + ALU
D4T + 3TC + NVP			
DDI + 3TC + EFV			
DDI + 3TC + NVP			If renal insufficiency and anemia: ABC + 3TC + ALU

AZT = zidozudine; 3TC = lamivudine; EFV = efavirenz; CNS = central nervous system; CBV = combivir; DDI = didanosine; NVP = nevirapine; DTG = dolutegravir; TRU = truvada; ABC = abacavir; TDF = tenofovir; FTC = emtricitabine, and ALU = aluvia. The third-line regimen comprised other alternative combination or salvage therapy. This was deployed in case of failure of both the standard first- and second-line regimens.

**Table 2:** Baseline characteristics and bivariate analysis of participants by BMI status at their first clinic visit (BMI-1) categorized as underweight, normal, and overweight/obesity (*N* = 531).

Characteristics		BMI at the first clinic visit (BMI-1)				
	Total	Underweight	Normal BMI	^††^Overweight/obesity	UOR	*P*-value
Age-1 at ART initiation [in years, (mean ± SD)]	41.4 ± 8.8	40.9 + 10.5	41.2 ± 8.6	42.2 ± 8.1	—	0.520
*Gender*						
Male, *n* (%)	163 (30.7)	45 (56.3)	99 (29.4)	19 (16.7)	1^†^	—
Female, *n* (%)	368 (69.3)	35 (43.7)	238 (70.6)	95 (83.3)	2.64	0.001^∗^
*CD4^+^ cells/mm^3^at first the clinic visit*						
Median (IQR)	132 (47–132)	99 (41–190)	133 (44–193)	150 (69–216)		0.030^∗^
*CD4^+^ cells/mm^3^ categories at the first clinic visit*						
≥350, *n* (%)	23 (4.3)	2 (2.5)	11 (3.6)	9 (7.9)	1^†^	—
Nadir (0–200), *n* (%)	408 (76.8)	62 (77.5)	266 (78.9)	80 (70.2)	0.24	<0.001^∗^
201–349, *n* (%)	100 (18.8)	16 (20.0)	59 (17.5)	25 (21.9)	0.33	<0.001^∗^

BMI = body mass index; overweight/obesity = aggregate of overweight, obesity and morbidly-obese; UOR = unadjusted odds ratio; ART = antiretroviral therapy; SD = standard deviation; IQR = interquartile rage; ^∗^*p* < 0.05; ^††^outcome of interest;^ †^reference group.

**Table 3:** ART recipients' BMI status at the first and last clinic visits at Princess Marina Hospital and Bontleng anti-retroviral therapy clinics in Botswana (*N* = 531).

BMI status (kg/m^2^)	Number and proportion of patients		McNemar test *p*-value
	First clinic visit, *n* (%)	Last clinic visit, *n* (%)	
Underweight	80 (15.1)	44 (8.3)	<0.001^∗^
Normal BMI	337 (63.5)	233 (43.9)	<0.001^∗^
Overweight	72 (13.6)	153 (28.8)	<0.001^∗^
Obesity	42 (7.9)	88 (16.6)	<0.001^∗^
Morbidly-obese	0 (0.0)	13 (2.4)	—
Overweight/obesity	114 (21.5)	241 (45.4)	<0.001^∗^
Obesity/morbidly-obese	42 (7.9)	101 (19.0)	<0.001^∗^

ART = antiretroviral therapy; BMI = body mass index; First clinic visit = when patients were enrolled in the study or before initiation on ART; Last clinic visit = after initiation on ART; ^∗^significant difference; Overweight/obesity = recipients with a BMI of overweight or any category of obesity.

**Table 4:** Characteristics and bivariate analysis of study participants by BMI at their last clinic visit (BMI-2) at Princess Marina Hospital and Bontleng anti-retroviral therapy clinics in Botswana categorized as underweight, normal and overweight/obesity (*N* = 531).

Characteristics	^†^BMI after patients' initiation on ART (BMI-2)				
	Total	Underweight	Normal BMI	^††^Overweight/obesity	UOR	*P*-value
Age after ART initiation [years, mean ± SD]	47.6 ± 9.6	50.1 ± 14.1	47.6 ± 9.6	47.3 ± 8.5	—	0.180

*ART regimen*						
Second/third-line, *n* (%)	208 (39.2)	13 (29.5)	101 (43.3)	88 (36.5)	1^†^	—
First-line, *n* (%)	323 (60.8)	31 (70.5)	132 (56.7)	153 (63.5)	1.2	0.250
Duration of exposure to ART (months, mean ± SD)	84.9 ± 29.7	91.7 ± 28.0	87 ± 30.1	81 ± 29.3	—	0.190

*Gender*						
Male, *n* (%)	163 (30.7)	28 (63.6)	89 (38.2)	45 (18.7)	1^†^	—
Female, *n* (%)	368 (69.3)	16 (36.4)	144 (61.8)	196 (81.3)	3.0	0.001^∗^

*CD4 cell count/mm^3^at last clinic visit*						
Median (IQR)	515 (310–691)	444 (270–597)	513 (375–686)	577 (416–732)	—	0.001^∗^

*CD4 cell count/mm^3^categories at last clinic visit*						
≥350, *n* (%)	421 (79.3)	22 (50.0)	183 (78.5)	216 (89.6)	1^†^	—
Nadir (0–200), *n* (%)	34 (6.4)	8 (18.2)	17 (7.3)	8 (3.3)	0.29	0.003^∗^
201–349, *n* (%)	76 (14.3)	14 (31.8)	33 (14.2)	17 (7.1)	0.27	0.001^∗^

*DRC*						
Absent, *n* (%)	442 (83.2)	40 (90.9)	201 (86.3)	189 (78.4)	1^†^	—
Present, *n* (%)	89 (16.8)	4 (9.1)	32 (13.7)	52 (21.6)	1.88	0.007^∗^

ART = antiretroviral therapy; BMI = body mass index; overweight/obesity = overweight or obesity and morbidly obese; UOR = unadjusted odds ratio; DRC = diabetes-related comorbidity; SD = standard deviation; ^∗^*p* < 0.05; ^†^reference group; ^††^outcome of interest.

**Table 5:** Predictors of overweight/obesity and factors independently associated with BMI changes among HIV patients after ART initiation at Princess Marina Hospital and Bontleng anti-retroviral therapy clinics in Botswana. Dependent variable: overweight/obesity (*N* = 531).

Independent variables	Number (%)	Unadjusted		Adjusted	
		OR	95% CI	OR	95% CI
Age-2 (years, mean ± SD)	47.6 ± 9.6	0.99	0.97–1.01	1.01	0.98–1.03

*ART regimen*					
Second- or third-line	208 (39.2)	1^†^	—	1^†^	—
First-line	323 (60.8)	1.2	0.86–1.74	1.22	0.82–1.79

Duration of exposure (months, mean ± SD)	84.9 ± 29.7	0.95	0.88–1.02	0.95	0.85–1.02

*Gender*					
Male	163 (30.7)	1^†^	—	1^†^	—
Female	368 (69.3)	3.00^∗∗^	2.00–4.45	2.84^∗∗^	1.83–4.42

*CD4 cell count/mm^3^*					
≥ 350	421 (79.3)	1^†^	—	1^†^	—
201-249	76 (14.3)	0.27^∗∗^	0.15–0.48	0.38^∗^	0.16–0.89
Nadir (0–200)	34 (6.4)	0.29^∗∗^	0.13–0.66	0.30^∗∗^	0.17–0.55

*DRC*					
Absent	442 (83.2)	1^†^	—	1^†^	—
Present	89 (16.8)	1.88^∗∗^	1.18–2.98	2.2^∗∗^	1.18–3.39

Age-2 = age at the last clinic visit; ART = antiretroviral therapy; DRC = diabetes-related comorbidity; OR = odd ratio; CI = confidence interval; ^∗^*p* < 0.05; ^∗∗^*p* < 0.001; Cox and Snell *R*^2^ = 0.12; Hosmer and Lemeshow *p* = 0.14; ^†^reference group.

## Data Availability

Data from which the findings of this study emanate are not publicly available to maintain patient confidentiality. The data include potentially identifying demographic and clinical care information. However, the data can be requested from the corresponding author who must first get permission from the management of the HIV clinics where the study was conducted before sharing.

## Abbreviations

3TC: Lamivudine
ABC: Abacavir
AIDS: Acquired immune deficiency syndrome
ALU: Aluvia
ANOVA: Analysis of the variance
AOR: Adjusted odds ratio
ART: Antiretroviral therapy
AZT: Azidothymidine, also known as zidovudine
BMI: Body mass index
CBV: Combivir
CD4: Cluster of differentiation 4
CD4-1: CD4 cell count at ART initiation
CD4-2: CD4 cell count at the time of data collection
CI: Confidence interval
CNS: Central nervous system
DDI: Didanosine
DRC: Diabetes mellitus-related comorbidities
DTG: Dolutegravir
EFV: Efavirenz
FTC: Emtricitabine
HIV: Human immunodeficiency virus
ICD-10: The 10^th^ revision of the international statistical classification of diseases and related health problems
IQR: Interquartile range
LDL-C: Low-density lipoprotein cholesterol (LDL-C)
NCDs: Noncommunicable diseases
NVP: Nevirapine
PLWH: People living with HIV
SD: Standard deviation
TDF: Tenofovir
TRU: Truvada
UNAIDS: Joint United Nations' Program on HIV/AIDS
UOR: Unadjusted odds ratios
Weight-1: Weight in kilograms at ART initiation
Weight-2: Weight in kilograms at the time of data collection
WHO: World health organization.

## Supplementary Materials

Supplementary Materials Standard first- and second-line ART regimens in Botswana.
