# Supplementary material for: Overweight and Obesity among Recipients of Antiretroviral Therapy at HIV Clinics in Gaborone, Botswana: Factors Associated with Change in Body Mass Index
Source: AIDS Res Treat. 2020 Jan 4;2020:8016791. doi: 10.1155/2020/8016791 (PMC7199624; doi:10.1155/2020/8016791)
Supplement: Supplementary Materials — Standard first- and second-line ART regimens in Botswana. [file 8016791.f1.docx]

**Supplementary material**

*Standard First- and Second-line ART Regimens in Botswana*

The Botswana National ART programme also called Masa was launched in 2002. Over the years the Ministry of Health has issued and updated anti-retroviral treatment guidelines based on WHO recommendations. First-line and second-line regimens have therefore been modified over the years from 2002-2016. Between 2002 and 2015, the period for this study, the treatment regimens were as per table below adapted from the 2012 and before 2016 treatment guidelines:

| First line | First line modifications | Second line | Second line modifications |
| --- | --- | --- | --- |
| AZT +3TC (CBV-combivir) + EFV  AZT + 3TC +NVP  AZT +DDI+ EFV  AZT+ DDI +NVP | TDF renal toxicity w/o CVD risk:  ABC/3TC/DTG  (If CVD rash: Consult HIV specialist)  CNS Toxicity and/or  Hepatic Toxicity:  TRU/DTG | TDF+FTC +ALU | AZT Anemia and/or  TDF Renal Toxicity: ABC/3TC/DTG |
| TDF+ FTC (or 3TC) + EFV  TDF+ FTC (or 3TC)+ NVP |  | CBV + ALU | If anemic ABC+ 3TC+ ALU |
| D4T + 3TC +EFV  D4T + 3TC +NVP  DDI +3TC +EFV  DDI+ 3TC +NVP |  | TDF+FTC=ALU | If renal insufficiency but no anemia: CBV+ALU  If renal insufficiency and anemia: ABC + 3TC+ALU |
